# The impact of genome editing on the introduction of monogenic traits in livestock

**DOI:** 10.1186/s12711-018-0389-7

**Published:** 2018-04-16

**Authors:** John W. M. Bastiaansen, Henk Bovenhuis, Martien A. M. Groenen, Hendrik-Jan Megens, Han A. Mulder

**Affiliations:** Wageningen University & Research Animal Breeding and Genomics, PO Box 338, 6700 AH Wageningen, The Netherlands

## Abstract

**Background:**

Genome editing technologies provide new tools for genetic improvement and have the potential to become the next game changer in animal and plant breeding. The aim of this study was to investigate how genome editing in combination with genomic selection can accelerate the introduction of a monogenic trait in a livestock population as compared to genomic selection alone.

**Methods:**

A breeding population was simulated under genomic selection for a polygenic trait. After reaching Bulmer equilibrium, the selection objective was to increase the allele frequency of a monogenic trait, with or without genome editing, in addition to improving the polygenic trait. Scenarios were compared for time to fixation of the desired allele, selection response for the polygenic trait, and level of inbreeding. The costs, in terms of number of editing procedures, were compared to the benefits of having more animals with the desired phenotype of the monogenic trait. Effects of reduced editing efficiency were investigated.

**Results:**

In a population of 20,000 selection candidates per generation, the total number of edited zygotes needed to reach fixation of the desired allele was 22,118, 7072, or 3912 with, no, moderate, or high selection emphasis on the monogenic trait, respectively. Genome editing resulted in up to four-fold faster fixation of the desired allele when efficiency was 100%, while the loss in long-term selection response for the polygenic trait was up to seven-fold less compared to genomic selection alone. With moderate selection emphasis on the monogenic trait, introduction of genome editing led to a four-fold reduction in the total number of animals showing the undesired phenotype before fixation. However, with a currently realistic editing efficiency of 4%, the number of required editing procedures increased by 72% and loss in selection response increased eight-fold compared to 100% efficiency. With low efficiency, loss in selection response was 29% more compared to genomic selection alone.

**Conclusions:**

Genome editing strongly decreased the time to fixation for a desired allele compared to genomic selection alone. Reduced editing efficiency had a major impact on the number of editing procedures and on the loss in selection response. In addition to ethical and welfare considerations of genome editing, a careful assessment of its technical costs and benefits is required.

**Electronic supplementary material:**

The online version of this article (10.1186/s12711-018-0389-7) contains supplementary material, which is available to authorized users.

## Background

Animal breeders have a long history in changing the genetic makeup of livestock. Until recently, this has only been possible through traditional selective breeding, which is a relatively slow process that accumulates desired alleles over many generations. In the 1980s and 1990s, the possibility of developing transgenic livestock seemed to offer an alternative approach of achieving selection response. By applying transgenic methodologies, a gene could be inserted into the genome of an organism at random positions, but this approach was hampered by technical difficulties and limitations and raised public concerns. So far, no transgenic livestock have been approved for human consumption, except transgenic salmon in the USA [1, 2].

The advent of genome editing (GE) has added new possibilities for altering genomes. New technologies such as CRISPR-Cas9 have considerably improved the level of efficiency and precision of modifying the genome compared to the transgenic methodology. Editing can be guided to any specific location in the genome and could be used to change genes so that they produce a different product or become non-functional genes. It has been suggested that CRISPR-Cas9 can be used to repair genetic defects, as demonstrated in mice [3], or to confer resistance to diseases, as reported in wheat and rice [4]. Progress in GE has also been made in cattle, sheep, pigs and goats [5–8], for instance to confer resistance to diseases [9] or introduce polledness (absence of horns) [10]. GE techniques hold much promise for the genetic improvement of livestock and have the potential to become the next game changer in animal and plant breeding.

Genome editing in livestock has primarily introduced alleles that affect monogenic traits [9, 10]. Improving a polygenic trait by promotion of alleles by genome editing (PAGE) in combination with genomic selection was simulated by Jenko et al. [11] and shown to increase response to selection after 20 generations by 1.08- to 4.12-fold compared to using genomic selection alone. In their simulation, it was assumed that genes and their effects were known without error. However, one of the major technical obstacles for implementation of GE in commercial breeding programs is the limited knowledge regarding the causative mutations that underlie the observed genetic variation. Therefore, PAGE may not be of interest for quantitative and complex traits in the near future because editing targets are lacking. Alternative approaches have been suggested that apply editing to many loci to simultaneously prove and use the effect of variants [12]. Designs for these schemes need to be developed and tested.

Introduction of alleles by GE may have several advantages compared to classical breeding strategies including genomic-assisted introgression. Classical introgression breeding strategies are difficult, costly and time-consuming, and also suffer from a lower genetic gain, linkage drag and increased inbreeding in the region surrounding the target gene [13, 14]. The use of genomic data can improve the efficiency of removing the donor genome [13–16] and further increase genetic gain [17, 18], but it has not led to the application of introgression in livestock.

When a monogenic trait is already present in the population, increasing the frequency of the desired allele from low starting values with classical breeding strategies is still difficult and results in increased inbreeding and decreased genetic gain in the total breeding goal [18, 19]. An example is the polled allele that is desired to breed dairy cattle without horns. Currently, horns are removed to prevent cattle from hurting each other or humans. However, dehorning is an invasive and painful procedure, which is expected to become further regulated or banned in some countries. In cattle, a single gene is responsible for polledness, but the corresponding allele is very rare in dairy cattle. It may easily take 20 to 30 years to reach fixation, and therefore this gene is a possible target for GE, since it would not have the disadvantages observed with selection provided that enough animals are edited successfully [11].

To date, it is not known how genomic selection and GE could be combined to reduce the number of animals to be edited, increase the allele frequency of a desired allele, and minimize the loss in genetic gain for other traits. Furthermore, the current efficiency of GE is low and the mortality of genome-edited zygotes is high [20]. Although CRISPR-Cas9 is a much more precise technique for GE than previous genetic modification techniques, edited animals may show off-target effects [21], which would result in culling selection candidates.

The aim of this study was to investigate the extent to which GE, in combination with genomic selection, could contribute to the change in frequency of an allele with a monogenic effect compared to genomic selection alone. We investigated the effects of the weight on the desired allele in the breeding goal, the rate of success of GE, and the survival rate of edited zygotes on the change in allele frequency using Monte Carlo simulation. Furthermore, the effects on genetic gain in the polygenic trait and on the rate of inbreeding were assessed. The benefits from having animals with the desired trait and the cost, in terms of number of animals to be edited, were compared.

## Methods

The aim of this study was to mimic a livestock breeding population that, historically, had been selected for a polygenic trait (or index of traits). Starting in generation 0, increasing the frequency of the desired allele of a monogenic trait, such as polledness in cattle [22, 23], was added to the breeding goal.

### Simulation of animals

**Fig. 1:**
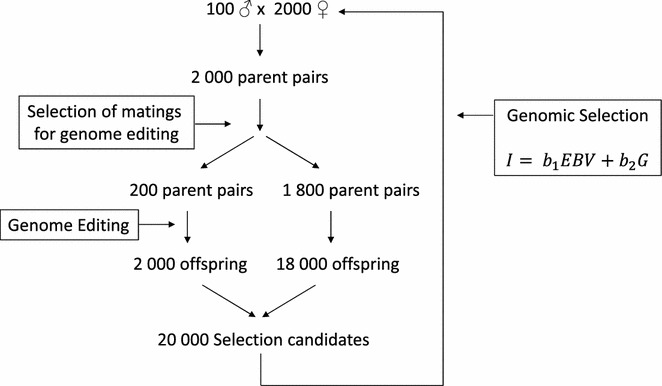
Schematic representation of the simulated breeding program

A breeding population was simulated with discrete generations consisting of 100 male parents and 2000 female parents per generation (Fig. 1). To produce the next generation, one male parent was randomly assigned to each female parent. Each mating produced 10 offspring, resulting in 20,000 animals per generation with random sexes assigned with 0.5:0.5 probabilities. Animals in the founder generation received a true breeding value (TBV) for the polygenic trait by drawing random values from a normal distribution with a mean of zero and a variance of 1 (N(0,1)) using the function rnorm()in R software [24]. TBV of the offspring in later generations were half the TBV of the sire plus half the TBV of the dam plus a Mendelian sampling effect drawn from a normal distribution N(0, 0.5(1 − 0.5(F_sire_ + F_dam_))). A genotype for the monogenic trait was assigned to all founders based on Hardy–Weinberg equilibrium proportions and a frequency for the desired allele of 0.01. A low starting frequency was assumed to allow comparison to selection scenarios without GE. The monogenic trait was controlled by a single gene with no effect on the polygenic trait, such as the dominant trait polledness in cattle [22, 23] or the recessive trait resistance to *Escherichia coli* F18 in pigs [25]. For these examples, the starting frequency of the desired allele would be higher than the 0.01 value that was chosen as the starting point for simulations.

In addition to the main scenario with 100 males, 2000 females and 10 offspring per mating, additional scenarios were simulated with population sizes that were similar to real breeding programs in dairy cattle, pigs, and fish. In dairy cattle, the numbers of males and females per generation and the number of offspring per female were 200, 600, and 16, respectively. These numbers were 50, 2000, and 20, and 120, 240 and 40, for pigs and fish, respectively. A “small” breeding program was also included with 20 males, 240 females, and 70 offspring per female. Other parameters for these species-specific designs were kept the same as in the main simulation scenario.

### Selection

Selection was simulated in two phases. The first five generations of selection were only on the estimated breeding value (EBV) of the polygenic trait. This first phase was used to reach equilibrium genetic gain, after reduction of the genetic variance due to the Bulmer effect [26]. Truncation selection was applied to choose 100 sires and 2000 dams with the highest EBV from the total population of 20,000 candidates. The default breeding program, without GE, was a scheme where selection was for a genomic EBV with a reliability of 0.5 (*r*^2^). The EBV was simulated by adding to the TBV a prediction error (PE) that was drawn from a normal distribution, N(0,$\frac{{1 - r^{2} }}{{r^{2} }}$), i.e. EBV = TBV + PE. Subsequently, the EBV were scaled to the proper variance by multiplying them with *r*^2^.

In the second phase, the same numbers of sires (100) and dams (2000) were selected from 20,000 candidates in each of the 20 generations by truncation selection on an index, $I = b_{1} {\text{EBV}} + b_{2} G$, where *b*_1_ and *b*_2_ were the index weights and *G* was the number of desired alleles for the monogenic trait. The EBV was calculated as in phase 1 and the genotypes for the monogenic trait were assumed known without error. Index weight *b*_1_ was fixed to 1 and *b*_2_ had a value of 0, 0.5, or 1000 (Table 1). With *b*_2_ = 0, selection was only on the EBV, i.e. genomic selection on the polygenic trait. With *b*_2_ = 1000, maximum emphasis was put on the monogenic genotype. The value of *b*_2_ = 0.5 was chosen empirically so that the change in frequency of the desired allele followed an intermediate pattern.

**Table 1:** Scenarios simulated

Method	*b*_2_^a^	*k* ^b^	*s* ^c^	N_edited_^d^
GS + GE	0	1	1	2000
	0.5	0.1, 0.2, 0.4, 0.6, 0.8, 1.0	0.05, 0.2, 0.4, 0.6, 0.8, 1.0	2000
	1000	1	1	2000

^a^Index weight for the monogenic trait genotype ($I = b_{1} {\text{EBV}} + b_{2} G$), same *b*_2_ values were used for GS

^b^Probability of successful editing

^c^Probability of survival for edited offspring

^d^Number of zygotes edited

### Genome editing

We assumed that GE of the gene that affects the monogenic trait was applied to zygotes during reproduction. After mate assignment, matings were selected for editing and the resulting zygotes were subjected to GE procedures and could lead to edited offspring. The genotype of the selected parents did not change. In the different scenarios, GE was applied to the zygotes of either none of the matings, 10% of the matings, or all matings. When editing was limited to 10% of the matings, we selected those that (1) had the smallest number of desired alleles for the monogenic trait in the parents and (2) the best parent average for *I*. Genome editing changed all undesired alleles in the offspring into the desired alleles. An editing success probability, *k*, was applied separately to each allele and was set to 1.00, 0.80, 0.60, 0.40, 0.20 or 0.10 in different scenarios (Table 1). A probability of surviving the editing procedure, *s*, was applied to each zygote that was subjected to editing and was set to 1.00, 0.80, 0.60, 0.40, 0.20 or 0.05 in different scenarios (Table 1). Editing success and editing survival were the result of a Bernoulli trial with, respectively, probabilities *k* or *s*. Values of *s* lower than 1.00 resulted in edited families having fewer offspring than families that were not selected for GE.

Currently, efficiency of GE is low, with one live edited offspring for 24 edited zygotes reported in the literature [20]. To mimic this low efficiency, an editing success probability *k* of 0.20 was combined with a survival probability *s* of 0.20 in order to have one live edited offspring for every 25 zygotes edited. These values were used in a GS + GE scenario with *b*_2_ = 0.5.

### Evaluation of scenarios

All scenarios were replicated 50 times. The number of editing procedures was counted at each generation as the number of zygotes that were genome-edited. Genome editing was applied only to families in which the parents carried at least one copy of the undesired allele. The genetic level in each generation was calculated as the average TBV and selection response was calculated as the difference in average TBV between the current and previous generation. Inbreeding of all individuals was calculated with the function calcInbreeding() of the package pedigree [27] in R [24]. The inbreeding level in each generation was calculated as the average inbreeding coefficient, and inbreeding rate was calculated as the difference in average inbreeding coefficient between the current and previous generation. Frequency of the monogenic allele and the variance of TBV were calculated per generation.

### Comparing costs and benefits

Choosing GE to increase the frequency of a desired allele will depend on, among other considerations, the costs and benefits of alternative approaches. The cost–benefit comparison was performed at a breeding horizon of 5 generations and at 20 generations. Costs of using GE were assumed to be mainly due to the editing procedures. The cumulative number of edited zygotes was used as a measure of costs. Benefits of using GE were calculated as the extra offspring that had the desired phenotype due to application of GE. Between scenarios, we compared the percentage of animals with the desired phenotype for the monogenic trait, assuming either dominant or recessive gene action.

Adding the monogenic trait to the index will increase the frequency of the desired allele but, at the same time, reduce selection response for the polygenic trait. The parameters assessed in generations 5 and 20 were the loss in selection response for the polygenic trait in genetic standard deviations and in months, assuming a generation interval of 2 years, the cumulative inbreeding, and the frequency of the desired allele. The loss in selection response was based on the difference in mean TBV of a specific scenario and the mean TBV in the same generation in the GS scenario with *b*_2_ = 0. The loss in selection response is presented in months by dividing this difference in mean TBV by the equilibrium response in generation 0 and multiplying by the generation interval of 24 months. The loss in selection response is also presented in genetic standard deviations using equilibrium genetic variance in generation 0.

## Results

After five generations of selection on only the EBV for the polygenic trait in phase 1, the equilibrium genetic variance (*σ*_*A*_^2^) and equilibrium selection response were reached with a genetic variance of 0.73 and a selection response per generation of 1.13. The allele frequency of the monogenic trait in generation 0 remained at the starting frequency, on average 0.01 ± 0.01. The desired allele of the monogenic trait was lost due to drift in 14 out of the 950 replicates across all scenarios (1.5%).

### Frequency of the monogenic allele

**Fig. 2:**
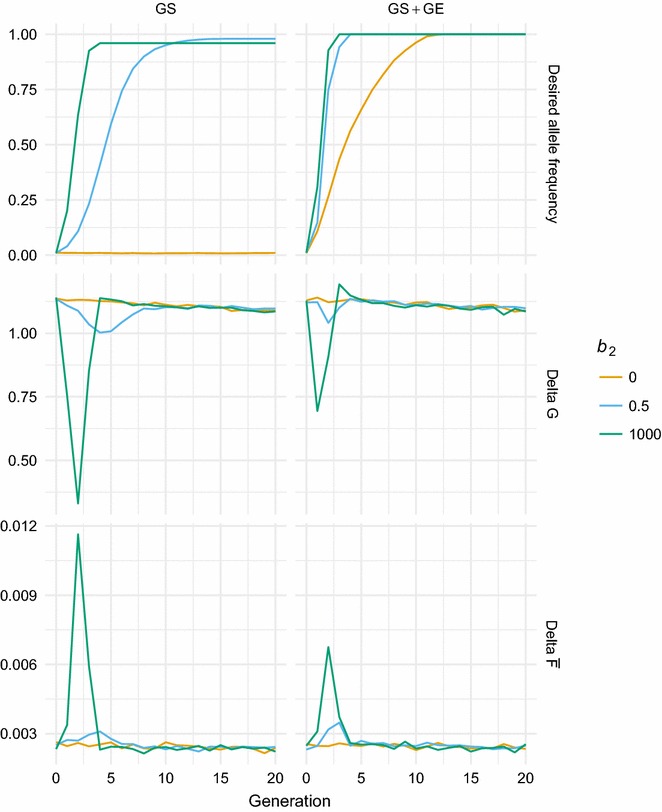
Response to selection. Frequency of the desired allele, change in *G*, and $\overline{F}$, in response to genomic selection (GS) and in response to genomic selection with genome editing (GS + GE), applying different weights (*b*_2_) on the desired allele of a monogenic trait

The time to fixation for the monogenic genotype was very short with the maximum weight on the monogenic genotype and very similar to that obtained with four generations by GS and three generations by GS + GE (Table 2; Fig. 2). With a moderate weight on the desired allele (*b*_2_ = 0.5), the time to fixation became very different between scenarios with 17 generations for GS and only five generations for GS + GE (Table 2). With a moderate weight on the desired allele, GE greatly reduced the time to fixation.

**Table 2:** Generations to fixation of monogenic trait

*b*_2_^a^	GS^b^	GS + GE^c^
0	inf	13
0.5	19^d^	5
1000	4^d^	3

^a^Index weight for the genotype of the monogenic trait

^b^Genomic selection only

^c^Genomic selection + genome editing

^d^Time to fixation in replicates in which the desired allele is not lost due to drift

In most scenarios, the desired allele became fixed (Fig. 2), with a few exceptions. The allele frequency did not change in the GS scenario with zero weight on the desired allele (*b*_2_ = 0). In the GS scenario with *b*_2_ > 0, the desired allele was fixed in all except three of the 100 replicates for which the desired allele was lost due to drift (Fig. 2). The GS + GE scenarios always resulted in fixation of the desired allele because even if the desired allele was lost due to drift, it was re-introduced by GE. With a zero weight, fixation was reached after 13 generations.

### Selection response for the polygenic trait

With *b*_2_ = 0, selection in phase 2 remained the same as in phase 1: genomic selection for the polygenic trait. Due to inbreeding, the selection response for the polygenic trait decreased from an equilibrium response of 1.13 in generation 0, to 1.09 in generation 20 (Fig. 2).

When maximum emphasis was given to the desired allele (*b*_2_ = 1000), a sharp decrease in the response for the polygenic trait was observed in generations 1, 2 and 3 (Fig. 2). In generation 2, the response was only 29% of the equilibrium selection response in generation 0. When combined with GE, a sharp decrease in selection response was still observed, but only in generations 1 and 2 (Fig. 2), and the lowest response still reached 62% of the equilibrium response in generation 0. Thus, loss in polygenic response due to selection for the monogenic trait was more than halved by GE.

With a moderate weight on the desired allele (*b*_2_ = 0.5), the reduced response lasted much longer, until generation 9, for the GS scenario (Fig. 2). In the GS + GE scenario, the response was reduced for three generations with *b*_2_ = 0.5, which is only one generation more than with the maximum *b*_2_ = 1000. With *b*_2_= 0.5, the minimum responses were similar with GS and GS + GE scenarios.

### Inbreeding

Inbreeding was calculated with the pedigree that traced back to the founders in generation -5. In generation 0, average inbreeding $\overline{F}$ was 0.9%. The increase in $\overline{F}$ was larger in generations in which the selection emphasis on the desired allele was strong. The biggest increase in $\overline{F}$ was seen in the same generation or one generation after the largest decrease in selection response for the polygenic trait (Fig. 2). Genome editing reduced selection pressure on the desired allele and therefore resulted in lower rates of inbreeding in the GS + GE scenario in the generations in which the desired allele was not fixed.

Without selection on the desired allele (*b*_2_ = 0), the inbreeding level was 2.2% in generation 5 (Table 3) and with moderate selection (*b*_2_ = 0.5), the inbreeding level in generation 5 was only slightly higher at 2.3 or 2.4%. These inbreeding levels in generation 5, with no selection or moderate selection on the desired allele, were very similar, regardless of whether GE was used or not (Table 3). With moderate or no selection pressure on the desired allele, inbreeding levels were the same after 20 generations of selection whether GS or GS + GE was applied. Only when all the selection emphasis was on the desired allele (*b*_2_ = 1000), did GE decrease the long-term additional inbreeding from 1.2% with GS to 0.6% with GS + GE (Table 4).

**Table 3:** Population parameters after five generations of selection

Method	*b*_2_^a^	Reduced response		Allele frequency^c^	$\overline{F}$ ^d^	Number^e^	Cumulative benefit^f^	
		*σ*_*A*_	Months^b^				Dominant (%)	Recessive (%)
	0	0	0	0.01	0.022	0	2.1	0.0
GS	0.5	− 0.57	− 8.9	0.59	0.024	0	43.6	11.7
	1000	− 1.95	− 30.2	0.96	0.035	0	84.2	62.9
	0	− 0.02	− 0.3	0.66	0.022	10,000	60.6	20.4
GS + GE	0.5	− 0.23	− 3.5	1.00	0.023	7072	84.3	69.4
	1000	− 0.79	− 12.2	1.00	0.028	3912	90.4	79.1

^a^Index weight for the genotype of the monogenic trait

^b^Months of selection response lost for the polygenic trait in generation 5, compared to the genetic level of genomic selection (GS) with *b*_2_ = 0

^c^Frequency of the desired allele in generation 5

^d^Mean inbreeding coefficient in generation 5

^e^Cumulative number of editing procedures over 5 generations

^f^Percentage of animals, cumulative over the 5 generations, with the desired phenotype when the desired allele is either dominant or recessive

**Table 4:** Population parameters after 20 generations of selection

Method	*b*_2_^a^	Reduced response		Frequency^c^	$\overline{F}$ ^d^	Number^e^	Cumulative benefit^f^	
		*σ*_*A*_	Months^b^				Dominant (%)	Recessive (%)
	0	0	0	0.01	0.058	0	1.9	0.0
GS	0.5	− 0.76	− 11.9	0.98	0.060	0	85.3	69.9
	1000	− 2.02	− 31.3	0.96	0.070	0	95.9	84.8
	0	+ 0.07	+ 1.05	1.00	0.058	22,118	89.6	74.0
GS + GE	0.5	− 0.11	− 1.7	1.00	0.060	7072	96.1	92.4
	1000	− 0.83	− 12.9	1.00	0.064	3912	97.6	94.8

^a^Index weight for the genotype of the monogenic trait

^b^Months of selection response lost for the polygenic trait in generation 20, compared to the genetic level of genomic selection (GS) with *b*_2_ = 0

^c^Frequency of the desired allele in generation 20

^d^Mean inbreeding coefficient in generation 20

^e^Cumulative number of zygotes edited over 20 generations

^f^Percentage of animals, cumulative over the 20 generations, with the desired phenotype when the desired allele is either dominant or recessive

Inbreeding was also assessed in species-specific scenarios in which numbers of males and females per generation were chosen to be close to those of real breeding programs. Inbreeding levels without selection on the desired allele were 4.9, 12.2, 11.7, and 31.4% in generation 20 for the cattle, pig, fish, and “small” breeding scenarios, respectively. In scenarios with all the selection emphasis on the desired allele, the long-term additional inbreeding was 1.2, 2.1, 3.2, and 4.9% with GS and 0.6, 1.1, 1.4, and 2.6% with GS + GE, respectively for the cattle, pig, fish and “small” breeding scenarios. The addition of GE reduced the long-term additional inbreeding by approximately half in all scenarios compared to GS alone.

### Costs and benefits

The cumulative benefit of increasing the frequency of the desired allele was measured as the percentage of animals that showed the desired phenotype, across all generations after generation 0. For a dominant trait, this included animals with the heterozygous and with the desired homozygous genotype, and for a recessive trait, this only included animals with the desired homozygous genotype. Scenarios were compared at generation 5 (Table 3) and generation 20 (Table 4).

In a situation where fixation of the desired allele is essential in the short term, all the weight can be put on the desired allele. With GS, fixation took four generations (Table 2) and incurred a loss in selection response for the polygenic trait of 31.3 months (Table 4). In this case, the number of animals that still showed the undesired phenotype before fixation was (100% − 95.9%) × 20 generations × 20,000 offspring = 16,400 animals for a dominant allele (Table 4). With GS + GE, the additional cost was due to the GE of 3912 zygotes while the loss in selection response was reduced by 59% from 31.3 to 12.9 months and the number of animals with undesired phenotypes was equal to 9600 (41% less than the 16,400 animals with GS).

With a moderate weight (*b*_2_ = 0.5), the desired allele was fixed with GS after 19 generations (Table 2), the long-term genetic loss after 20 generations was 11.9 months (Table 4), and 58,800 animals had the undesired phenotype (dominant desired allele). With GS + GE, the additional cost was due to GE of 7072 zygotes while the loss in selection response was reduced to 1.7 months (86% less than 11.9 months with GS). The number of animals with the undesired phenotype decreased to 15,600 (73% less than the 58,800 with GS) for a dominant effect. The number of animals with the undesired phenotype decreased by approximately fourfold with GS + GE compared to GS.

### Editing success rate and editing survival

**Fig. 3:**
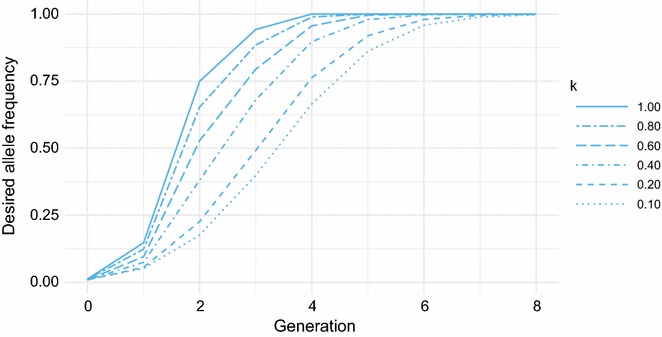
Response in frequency of the desired allele with reduced editing success. Allele frequency under genomic selection with genome editing (GS + GE) for different levels of editing success probability (*k*)

Both editing success rate (*k*) and editing survival (*s*) were assumed to be equal to 1.00 for all scenarios presented so far. When *k* was reduced from 1.0 to 0.10 (scenario GS + GE with *b*_2_ = 0.5), the required number of editing procedures almost doubled (Table 5), the selection response fell behind by 5 months (Table 5), and the time to fixation doubled from four to eight generations (Fig. 3).

**Table 5:** Long-term impact of success rate of genome editing

Success rate^a^	Months^b^	Procedures^c^	Cumulative benefit^d^	
			Dominant (%)	Recessive (%)
1.00	0.0	7072	96.1	92.4
0.80	+ 0.7	8184	95.5	91.0
0.60	− 2.4	9006	94.6	89.1
0.40	− 2.1	10,418	93.2	86.8
0.20	− 5.3	12,299	90.9	83.3
0.10	− 5.2	13,162	89.6	81.3

Impact of editing success rate under moderate selection intensity for the desired allele combined with genome editing

^a^Success rate of editing an undesired allele into the desired allele

^b^Long-term loss (measured at generation 20) in months of selection response for the polygenic trait, compared to a success rate of 1.00

^c^Total number of editing procedures

^d^Percentage of animals with the desired phenotype, cumulative over 20 generations, when the desired allele is either dominant or recessive

**Fig. 4:**
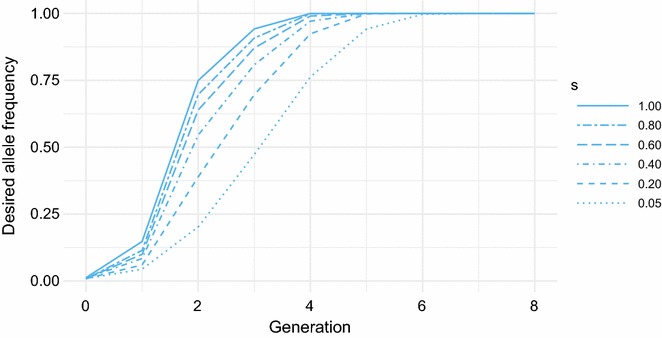
Response in frequency of the desired allele with reduced editing survival. Allele frequency under genomic selection with genome editing (GS + GE) for different levels of editing survival (*s*)

When *s* was reduced from 1.0 to 0.05, the increase in number of editing procedures was smaller than with reduced *k*, around 50% more instead of 100% (Table 6), and the time to fixation also increased by 50% from four to six generations (Fig. 4). When *s* was low, surviving zygotes were always edited and thus likely to be selected. When *k* was low, non-edited offspring could be selected over their edited full-sibs, when their EBV for the polygenic trait was sufficiently high.

**Table 6:** Long-term impact of survival rate of genome editing

Editing survival^a^	Months^b^	Procedures^c^	Cumulative benefit^d^	
			Dominant (%)	Recessive (%)
1.00	0.0	7072	96.1	92.4
0.80	− 1.5	7737	95.5	91.6
0.60	− 2.8	8109	95.1	90.8
0.40	− 5.1	8501	94.6	89.5
0.20	− 7.4	9514	93.2	87.5
0.05	− 12.8	11,071	90.5	83.6

Impact of editing survival rate under moderate selection intensity for the desired allele combined with genome editing

^a^Survival rate of zygotes subjected to editing

^b^Long-term loss (measured at generation 20) in selection response for the polygenic trait, compared to complete survival

^c^Total number of editing procedures

^d^Percentage of animals with the desired phenotype, cumulative over 20 generations, when the desired allele is either dominant or recessive

While reduced *k* had more impact on the number of zygotes that needed to be edited, reduced *s* had more impact on the loss in selection response. For *k* or *s* of 20%, the loss in selection response was 5.3 months versus 7.4 months, respectively (Tables 5 and 6).

### Efficiency of genome editing

To mimic the current low efficiency of GE [20], a *k* of 0.20 was combined with a *s* of 0.20 in order to reach one successfully edited zygote for 25 edited zygotes. When these values were used in the GS + GE scenario with *b*_2_ = 0.5, fixation of the desired allele was obtained after nine generations with 12,144 zygotes edited (Table 7). In comparison to the GS scenario without GE, the number of animals with the undesired phenotype before fixation was 26% less, and the long-term loss in selection response was 28% larger. In comparison to complete editing efficiency, 72% more zygotes needed to be edited and the loss in selection response was 8 times higher (Table 7). The impact of reduced efficiency was relatively larger on the loss of selection response than on the increased number of edited zygotes. With an efficiency of 4%, GE may enhance the increase in frequency of the desired allele compared to genomic selection, but increased the reduction in selection response due to reduced selection intensity.

**Table 7:** Population parameters in generations 5 and 20 with an editing success of 0.20 and a survival rate of 0.20

Generation^a^	Reduced response		Frequency^c^	$\overline{F}$ ^d^	Procedures^e^	Cumulative benefit^f^	
	*σ*_*A*_	Months^b^				Dominant (%)	Recessive (%)
5	− 0.88	− 13.6	0.86	0.023	9974	56.6	26.0
20	− 0.99	− 15.3	1.00	0.059	12,144	89.1	81.2

^a^Index weight for the genotype of the monogenic trait was *b*_2_ = 0.5

^b^Months of selection response lost for polygenic BV compared to the genetic level of genomic selection (GS) with *b*_2_ = 0 in the same generation

^c^Frequency of the desired allele

^d^Mean inbreeding coefficient

^e^Cumulative number of editing procedures up to the current generation

^f^Percentage of animals with the desired phenotype, cumulative up to the current generation, when the desired allele is either dominant or recessive

## Discussion

The objective of this study was to investigate how GE in combination with genomic selection could accelerate the increase in frequency of the desired allele for a monogenic trait compared to genomic selection alone. Assuming 100% accuracy and survival allowed an assessment of the potential of the technology. We observed a strong favourable impact of GE on time to fixation, loss in polygenic response, and number of animals that had the undesired phenotype before the desired allele was fixed. The main results are summarized in Table 8.

**Table 8:** Summary of results on the impact of genome editing and of reduced editing efficiency

Efficiency^a^ (%)	Measure	GS^b^	GS + GE^c^	Impact of GE^d^ (%)	Impact of efficiency^e^ (%)
100	Time to fixation (generations)	19	5	− 74	
	Loss in polygenic trait response (months)	− 11.9	− 1.7	− 86	
	inbreeding level in generation 20 (F)	0.06	0.06	0	
	Undesired phenotypes (N)	58,800	15,600	− 73	
	Editing procedures (N)		7072		
4	Polygenic trait response (months)		− 15.3		+ 800
	Editing procedures (N)		12,144		+ 72

Values from scenarios with moderate selection emphasis on the desired allele (*b*_2_ = 0.5)

^a^Percentage of edited zygotes surviving to reproduction age, 100% from *k* = 1, *s* = 1, 4% from *k* = 0.2, *s* = 0.2 (Table 1)

^b^Values from scenario with genomic selection only

^c^Values from scenario combining genomic selection and genome editing

^d^Percentage change between columns GS and GS + GE

^e^Percentage change from GS + GE with 100% efficiency to GS + GE with 4% efficiency

### Breeding programs with genome editing

In this study, the opportunities of applying GE to increase the frequency of the desired allele of a monogenic trait were evaluated. Earlier results of simulations of breeding with GE showed that polygenic traits could be improved by using PAGE [11]. An important hurdle to overcome for applying PAGE to quantitative traits and for increasing allele frequencies of monogenic traits is the availability of target mutations to edit. Only a small number of causal mutations are known that affect quantitative traits, and also the number of known mutations for monogenic traits is still limited. Making the application of GE cost-effective with a small number of targets that have known effects is more likely for a monogenic trait than for a quantitative trait nucleotide (QTN) that affects a polygenic trait. With PAGE, an increased selection response of 4 to 8% was reached by making a single edit in each sire, provided that all the QTN were known and different targets could be used in different sires [11]. Alternatively, GE could be applied to many loci to simultaneously prove and use the effect of variants [12]. With this approach, the benefits from GE can be much larger than those shown in simulation studies to date, in which a limited number of known QTN were assumed available. When multiple edits can be made to the same zygote at low cost, the integrated testing and use of edits may make GE on QTN of small or uncertain effects cost-effective. Designs for these schemes need to be developed and tested and could give better results as “unproven” variants may just be neutral.

Having only one single target may still be beneficial if its value for the population is large, as for instance when a single gene can confer resistance to disease. A monogenic trait, such as polledness, can also be highly relevant in view of the costs and the required changes in animal welfare regulations for dehorning of cattle. A single gene-editing target that is responsible for a small percentage of the genetic variance of a quantitative trait may have limited value. The money needed to perform GE on such a target may result in a bigger return when it is spend on additional genotyping or phenotyping to increase selection response.

### Inbreeding

In comparison to current breeding programs, the observed rate of inbreeding in these simulations was low, i.e. 0.25% per generation, mainly because of the large number of parents, in particular, the number of males per generation (100) was larger than that commonly used in livestock breeding programs. The level of inbreeding increased when emphasis of selection on the desired allele increased (higher *b*_2_), but not when GE was added to the scenarios. With *b*_2_ = 1000, inbreeding rate was actually lower in GS + GE compared to GS scenarios (Tables 3 and 4). An increase in inbreeding rate may be observed when adding PAGE to GS in scenarios in which a small number of sires is edited [11]. In our simulations, edits were made in zygotes from many matings, which requires more editing procedures, but in this way, it actually reduced inbreeding because the desired allele becomes available in many more families.

To test the impact on inbreeding with smaller population sizes, four species-specific scenarios were evaluated. In all those scenarios, the average inbreeding rate after 20 generations was similar in GS and GS + GE scenarios, except with *b*_2_ = 1000. Even with a realistic number of parents per generation, inbreeding rate was still relatively low for dairy cattle, pig and fish breeding scenarios, ranging from 4.7 to 12.2% in generation 20. This low inbreeding rate in our simulations is probably because contributions of all parents were assumed equal. Therefore, a scenario with a small breeding program was included that resulted in an average inbreeding level of 31.4% in generation 20. Additional inbreeding from selection on the desired allele increased with a smaller effective population size but the benefit of using GS + GE instead of GS also increased, such that the additional inbreeding was halved in all scenarios by adding GE.

### Selection index

The polygenic breeding value and the frequency of the monogenic allele were selected for simultaneously by combining the polygenic EBV and monogenic genotype in an index with fixed index weights. This index was the same for scenarios with and without GE. In these simulations, the trade-off between changing the allele frequency of the monogenic trait and the genetic progress for the polygenic trait was not optimized against genetic progress or inbreeding. Instead, three very different scenarios were included depending on the relative emphasis that was given to the frequency of the desired allele.

For the intermediate scenarios with *b*_2_ = 0.5, optimization of the allele frequency trajectory could reduce the loss in genetic progress, and also the rate of inbreeding [28–31]. The weights for the polygenic trait and the desired allele can be optimized given the starting allele frequency and the time at which fixation is desired. Optimization of the change in frequency of the desired allele with selection can reduce the disadvantage of GS scenarios in comparison to GS + GE.

### Alternatives for genome editing

Introducing new alleles or increasing the frequencies of desired alleles does not necessarily require GE. Alternatives are introgression and selection for the desired alleles. Disadvantages of introgression are potentially a lower genetic level of the donor breed and lower genetic gain, linkage drag and increased inbreeding in the region surrounding the target gene [13, 14]. In our simulations, we assumed that the desired allele was already present in the population at a starting frequency of 0.01. The carriers of the desired allele were chosen at random and therefore, on average, at the same genetic level as non-carriers. This was an advantage compared to introgression from another (inferior) breed. This advantage will also apply when the desired allele needs to be introduced by GE. Moreover, by choosing the animals with the highest EBV as targets for GE, as was done in the GS + GE scenarios, the desired allele can actually become favourably correlated with the polygenic trait. This is the opposite effect from linkage drag as observed with introgression and selection, for which a negative correlation between the polygenic and the monogenic traits may arise because of gametic phase disequilibrium [26]. Genome editing offers clear benefits compared to alternatives when introducing new alleles or increasing allele frequencies from low values.

### Challenges with genome editing

Genome editing technology is being developed and tested in animals [6–8] but which procedures will be applied in breeding programs remains to be determined. Depending on the species, the use of reproductive technology is at different stages of implementation. In dairy cattle, in vitro production of embryos (IVP) and multiple ovulation and embryo transfer (MOET) are commonly used in breeding programs, and applying GE during IVP may be relatively easy to implement. In this case, the parents may be selected as candidates to produce edited offspring, as was simulated in this study. An alternative, as simulated by Jenko et al. [11], is to select the best animals based on their breeding value and apply GE to these elite animals. Editing these elite animals requires a cloning procedure to produce an edited copy of the selected animals such as somatic cell nuclear transfer [20], unless the same embryo can be genotyped and selected, and subsequently edited before developing into an offspring. This procedure could take place in vitro by selecting the best embryos based on their genomic EBV and subsequently using cells from the same embryo to make edited clones. The generation interval could increase if a cloning step is needed, but this can be kept to a minimum if the whole procedure of genotyping, genomic selection, and editing can be done in vitro.

In all cases, in vitro reproduction is an essential step to apply GE. Although in vitro reproduction techniques are frequently used in cattle, these techniques are much less developed in other major agricultural species such as pigs and chickens. Moreover, if editing of individuals is desired after obtaining their EBV, instead of editing the offspring of selected parents, the use of cloning somewhere in the process may be necessary.

Even when in vitro reproduction is an established technology, there are a number of technical issues to be solved with GE before large-scale application is feasible. Currently, the probability of obtaining a live edited animal from applying GE is not high. Survival of edited zygotes is one important factor, but also the technical accuracy of GE needs to be considered. Genome editing may result in mosaics in which, part of a tissue is edited and other parts are not. In addition, GE may lead to off-target effects, i.e. changes in the genome at other positions than those intended. The occurrence of mosaics and off-target effects can be considered as an increase in mortality, as simulated in this study, assuming that mosaicism and off-target effects are either lethal or that the animals are not used for breeding for ethical or safety reasons. However, while direct mortality is a clear outcome of a procedure, excluding mosaic embryos or embryos with off-target edits requires their identification, which will be challenging. Strategies to screen for these effects are needed to avoid them going undetected [32–35].

A final challenge with GE is the uncertainty about future acceptance of application of the technique in animal breeding. The legal framework for its application is not clear everywhere, which may limit the efforts put into research and potential applications. Some methods of application require cloning of animals, which is not allowed in several countries. Clearly, an ethical debate is needed on when and why the application of GE in animal breeding is desirable. The potential costs and benefits presented in this paper can contribute to this debate.

### Opportunities with genome editing

When frequency of a monogenic allele is increased by GE alone, and GE has no detrimental effects such as reduced survival, a breeding program would not suffer from a lower selection response for the polygenic trait. For breeding programs that supply different markets (with potentially different competitors), this allows compliance with regulations that apply for specific markets, for instance where dehorning is not allowed, without reducing the competitiveness in other markets that do not impose these restrictions.

Determining the benefits of a technology is not a simple calculation, since values cannot always be captured on the same scale. Here, we made an attempt to compare such values for polledness in cattle using the results in Table 3, for which the impact of different scenarios was evaluated after five generations of breeding. Over the course of five generations, our simulated population counted 100,000 new-born animals. The basic scenario was GS with *b*_2_ = 0, where no effort was made to reduce polledness. In this scenario, 97.9% of calves, or 97,900 animals, were born with horns and would have to be de-horned, which is a costly and painful procedure for the calves. Assuming that the cost of dehorning is €10 per animal, the total cost of dehorning in our simulated population amounts to almost one million € after five generations. When applying GE without selection in scenario GS + GE with *b*_2_ = 0, an additional 58.5% (60.6–2.1%) of the 100,000 new-born animals would have the polled phenotype, resulting in €585,000 saved after five generations. The associated cost was the application of GE in 10,000 zygotes. In this scenario, there was no negative impact on selection response or on inbreeding, so the breakeven cost for applying GE would have been €58.50 per zygote. In many countries, the national population of dairy cattle is much larger and the 100 selected males would be used to serve a much larger cow population, in which case the breakeven cost of an edited zygote would become much higher. With a cow population of 1 million animals and when evaluating costs and benefits after five generations, the breakeven cost for editing one zygote would be €2925.

### Editing targets

Genome editing has the potential to create a major change in how we perceive and implement genetic improvement of livestock and other species. While animal genetics research has put a lot of effort in the discovery and investigation of the effects of genomic variation on phenotypes, its implementation in breeding programs has been minimal. When GE becomes a cost-effective and accepted technology, the use of individual variants in breeding programs may finally become important. Such promises were also made for marker-assisted selection, but reality proved more difficult. The advantages of GE are that it does not rely only on variants that segregate in the target population, and, in case of a low allele frequency, it does not depend on the small number of animals in which the variant is found.

However, a drawback that GE shares with marker-assisted selection is that in order to make the best use of it, the targets to edit must be known. Among the targets described in the literature, are alleles of the *RELA* gene, which were shown to have a role in the resistance to African swine fever in warthog [36]. Introduction of these alleles by GE into domestic pigs was recently reported [37]. Another example is a deletion in the *prolactin receptor* gene that determines the slick coat and heat tolerance traits in Senepol cattle [38]. Introduction of this allele into other cattle breeds by GE has been suggested [38].

While there are some examples of variants that can be targets for GE, such as those above or the polled locus used here, the number of known targets of interest to breeding schemes is currently limited. Thus, there is a renewed need for identifying causal mutations in the genome and for predicting their effect on phenotypes. The number of GWAS studies in animals is very large [39], but the resulting knowledge about causal mutations is so far limited.

While it remains difficult to determine causal effects, variants that are present in rare breeds or locally-adapted populations will become more important. In the past, these variants were studied, but their introduction into commercial breeding populations was not considered due to the issues with introgression that were discussed earlier. With GE, all these variants become of interest for breeding populations, independent of their current genetic background.

## Conclusions

In our simulation, we showed that GE strongly decreased the time to fixation for the desired allele compared to genomic selection alone. The loss in selection response in the polygenic trait was much smaller, up to seven-fold, with the addition of GE. The same level of inbreeding was observed with or without GE, except when all the selection emphasis was placed on the monogenic trait; then GE reduced long-term inbreeding. Combining GE with moderate selection emphasis for the desired allele reduced by about four-fold the number of animals with the undesired phenotype over all generations. With a realistic editing efficiency of 4%, the number of required editing procedures increased by 72% and the loss in selection response increased by 800%. With low efficiency, loss in selection response was 29% more compared to genomic selection alone. In addition to ethical and welfare considerations, a careful assessment of the technical costs and benefits of GE in commercial livestock is required.

## Additional file

**Additional file 1.** Simulation_genome_editing.R. This file presents the simulation program in R to create the simulation scenarios presented.
